# Nicotinic acid inhibits glioma invasion by facilitating Snail1 degradation

**DOI:** 10.1038/srep43173

**Published:** 2017-03-03

**Authors:** Jiejing Li, Jiagui Qu, Yu Shi, Mark Perfetto, Zhuxian Ping, Laura Christian, Hua Niu, Shuting Mei, Qin Zhang, Xiangcai Yang, Shuo Wei

**Affiliations:** 1 Department of Clinical Laboratory, The Affiliated Hospital of KMUST, Medical School, Kunming University of Science and Technology, Kunming 650032, China; 2 Department of Clinical Laboratory, Children’s Hospital of Chongqing Medical University, Chongqing 400014, China; 3 Ministry of Education Key Laboratory of Child Development and Disorders; Chongqing Key Laboratory of Pediatrics; Chongqing Key Laboratory of Translational Medical Research in Cognitive Development and Learning and Memory Disorders, Chongqing 400014, China; 4 Department of Biology, West Virginia University, Morgantown, WV 26506, United States; 5 Department of Biological Sciences, University of Delaware, Newark, DE 19716, United States; 6 Department of Gerontology, First People’s Hospital of Yunnan Province, Kunming 650032, China

## Abstract

Malignant glioma is a formidable disease that commonly leads to death, mainly due to the invasion of tumor cells into neighboring tissues. Therefore, inhibition of tumor cell invasion may provide an effective therapy for malignant glioma. Here we report that nicotinic acid (NA), an essential vitamin, inhibits glioma cell invasion *in vitro* and *in vivo*. Treatment of the U251 glioma cells with NA *in vitro* results in reduced invasion, which is accompanied by a loss of mesenchymal phenotype and an increase in cell-cell adhesion. At the molecular level, transcription of the adherens junction protein E-cadherin is upregulated, leading to accumulation of E-cadherin protein at the cell-cell boundary. This can be attributed to NA’s ability to facilitate the ubiquitination and degradation of Snail1, a transcription factor that represses *E-cadherin* expression. Similarly, NA transiently inhibits neural crest migration in *Xenopus* embryos in a Snail1-dependent manner, indicating that the mechanism of action for NA in cell migration is evolutionarily conserved. We further show that NA injection blocks the infiltration of tumor cells into the adjacent brain tissues and improves animal survival in a rat model of glioma. These results suggest that NA treatment may be developed into a potential therapy for malignant glioma.

Malignant glioma is a type of tumor that derives from the glial cells in the nervous system, including those of neural crest origin^1^,^2^. Patients commonly succumb to this deadly disease within 5 years upon being diagnosed^3^. Glioma is classified into four distinctive pathological grades according to the World Health Organization (WHO). Among them, grade IV, also called glioblastoma multiforme (GBM), is recognized clinically as the most frequent and malignant category^4^. Currently, therapeutic strategies involve three approaches, which consist of maximal tolerable surgical resection paired with radiation and chemotherapy. The combination of these therapies is able to add only months of additional survival. The diffuse invasion of tumor cells into the surrounding brain tissues imparts the major challenge for therapy. Although early radical surgical interventions attempt to remove the entire affected brain hemisphere, patients are usually subjugated to cancer cells that had crossed into the other hemisphere^5^. Even now, with advanced microsurgical techniques, recurrence is still often inevitable. Glioma typically reoccurs within 1–2 cm of the primary tumor^6^. Hence, a primary challenge is to prevent glioma cells from uncontrolled migration and subsequent infiltration into other brain regions.

Cell migration is a finely tuned biological process that often involves epithelial–mesenchymal transition (EMT). EMT was initially defined in the early 1980s by Elizabeth Hay^7^, who described changes from epithelial to mesenchymal phenotype in the primitive streak of chick embryos. EMT is a process that is associated with remarkable changes in cell adhesion, polarity and migratory properties. During EMT, epithelial cells lose their adhesion and polarity, reorganize their cytoskeleton, and undergo changes in cell signaling that alter cell shape and reprogram gene expression. EMT is typically characterized by upregulation of mesenchymal markers such as Snail1 and downregulation of epithelial markers such as E-cadherin. This process has been shown to be of critical importance to normal developmental processes such as mesoderm and neural crest migration^8^,^9^. Interestingly, similar mechanisms are utilized in disease processes including wound healing, fibrosis and tumor metastasis^10^,^11^,^12^. Notably, EMT and EMT-like processes confer tumor cells with the ability to migrate, invade, and adopt stem cell-like properties that largely account for immunosuppression and tumor recurrence^13^,^14^. Therefore, suppressing EMT should contribute to cancer therapy in multiple facets.

Nicotinic acid (NA), a member of the vitamin B family, is well known for its functions in the treatment and prevention of atherosclerosis. NA is one of the most effective agents that provide protection against cardiovascular risk factors by decreasing low-density and very low-density lipoprotein levels, while simultaneously increasing high-density lipoprotein levels^15^. Mechanistically, NA has been shown to downregulate cyclic adenosine monophosphate, the major intracellular mediator of pro-lipolytic stimuli, thereby decreasing cellular levels of free fatty acids^16^. Recently, we and others reported that NA is also able to regulate intracellular calcium levels. Ma *et al*. demonstrated that a high concentration (50 mM) of NA can regulate the activities of heat-sensitive capsaicin receptors TRPV1–4, which are non-selective calcium-permeable cation channels. Meanwhile, we found that NA causes a transient reduction but subsequent elevation in free intracellular [Ca^2+^] in NIH 3T3 cells. Treatment with over 30 mM NA further leads to disassembly of the cytoskeleton in 3T3 cells and inhibition of intracellular melanosome transport in *Xenopus* embryos, effects that can be partially attributed to increased intracellular [Ca^2+^]^17^,^18^,^19^.

In the current study we assessed the effects of NA on the behavior of glioma cells. We found that NA inhibits the invasion of U251 GBM cells by facilitating the ubiquitination and degradation of Snail1, a transcription factor that promotes EMT. This mechanism is likely conserved through evolution, as NA also downregulates Snail1 levels and delays neural crest migration in *Xenopus* embryos. We also show that NA treatment inhibits the invasion of C6 glioma cells allografted in the rat brain and improves survival of these rats. Based on these data, we propose that NA treatment may be developed into an effective therapeutic method for malignant glioma.

## Results

### *In vitro* NA treatment causes loss of mesenchymal phenotype and reduced invasion of U251 cells

**Figure 1:**
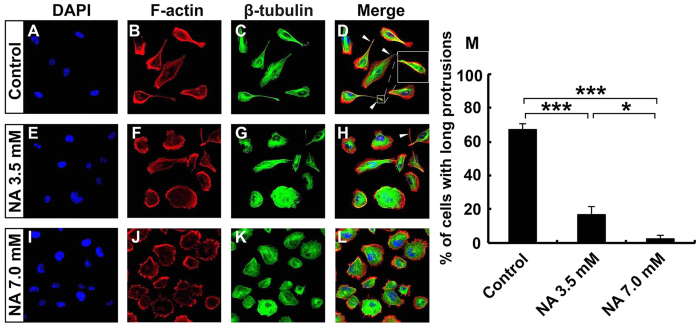
U251 cells lose mesenchymal phenotype upon NA treatment. U251 cells were incubated with PBS (control) or the indicated concentration of NA for 8 hr. Rhodamine phalloidin labeling for F-actin (red), immunocytochemistry for β-tubulin (green) and DAPI labeling for nuclei (blue) were carried out as described in Methods. White arrowheads denote the long protrusions that consist mainly of microtubules, and the inset in (**D**) shows an amplified image of the indicated protrusion. The percentage of cells with this type of long protrusions was calculated for each treatment and summarized in (**F**). *P < 0.05; ***P < 0.001.

We reported previously that high concentration of NA is able to disrupt cytoskeletal structures in 3T3 cells^17^. To assess if NA has any potential effects on malignant glioma, we treated cultured U251 GBM cells with various concentrations of NA. High concentrations of NA (14 mM and above) detached U251 cells and led to apoptosis (Yang X. *et al*., manuscript in preparation). By contrast, up to 7.0 mM NA did not cause any detectable cell death even after a prolonged period of treatment, as manifested by low annexin V and propidium iodide (PI) staining in the majority of cells with or without NA treatment (20 hr; Fig. S1A-S1D). However, we noticed a significant change in cell morphology with increasing concentrations of NA. The untreated U251 cells were mostly elongated and mesenchymal-like (Fig. 1A–D). Staining for F-actin and β-tubulin reveals that, similar to U87 GBM cells^20^, U251 cells often formed long protrusions that are primarily made of microtubules but also contain some actin filaments (white arrowheads in Fig. 1D,H; inset in Fig. 1D). Upon treatment with 3.5 mM NA, some of the U251 cells became rounded (Fig. 1E–H), whereas 7.0 mM NA caused most cells to lose the mesenchymal phenotype and long protrusions. Such a morphological change was accompanied by alterations in cytoskeletal structure: long F-actin stress fibers were significantly reduced, and microtubules became more uniformly distributed throughout the cells (Fig. 1I–L).

**Figure 2:**
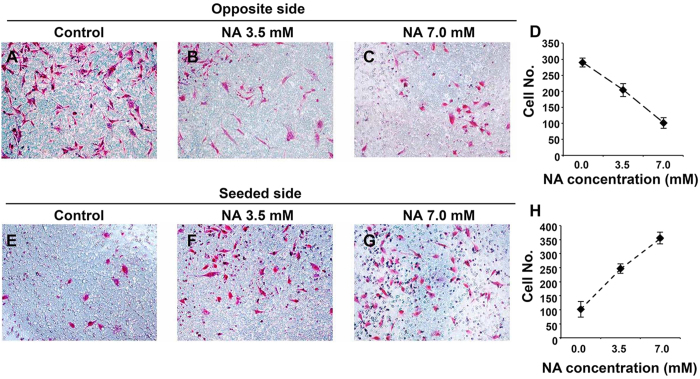
NA treatment inhibits U251 cell invasion *in vitro*. Transwell assays were carried out for U251 cells incubated in PBS (control) or the indicated concentration of NA as described in Methods. Images of cells (stained with Giemsa) that invaded through the matrigel (**A–C**) or remained on the seeded side (**E–G**) in a representative experiment are shown, and results of 15 different regions in 3 independent experiments (5 regions per experiment) are summarized in (**D**) and H for the opposite and seeded sides, respectively.

Although most U251 cells treated with NA still retained their actin-based filopodia, the significant reduction of cells with long protrusions that consist mainly of microtubules (Fig. 1M) suggests that NA may interfere with the migration/invasion of U251 cells, as the motility of GBM cells has been shown to be independent of actin polymers but dependent on microtubule assembly^20^. To assess this possibility, we performed Boyden chamber invasion assays. The invasive ability of U251 cells was considerably decreased with increasing concentrations of NA, as indicated by fewer cells that migrated through the matrigel (Fig. 2A–D) and more cells that were retained on the original seeded side (Fig. 2E–H). Thus NA inhibits the invasive ability of U251 cells, a main feature of GBM cells that contributes to high lethality. We further tested if NA affects the invasion of other types of cancer cells. As shown in Fig. S2A-S2H, 3.5 and 7.0 mM NA also reduced the invasive abilities of U87 GBM cells and B16F10 melanoma cells, suggesting that NA’s effect on cell invasion is not limited to U251 cells.

### NA upregulates cell-cell adhesion by promoting Snail1 ubiquitination and degradation in U251 cells

**Figure 3:**
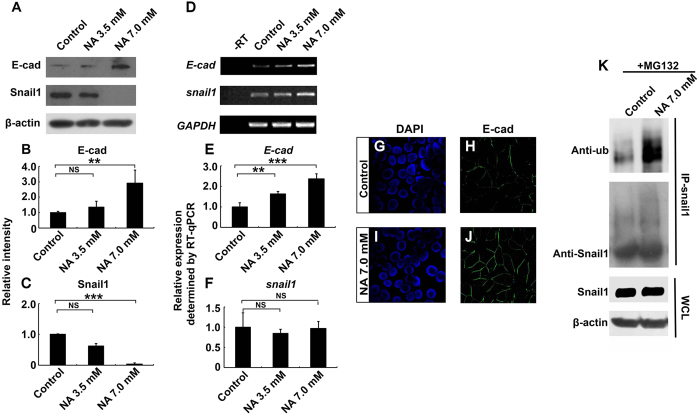
NA upregulates *E-cadherin* expression by promoting ubiquitination and degradation of Snail1. U251 cells were treated with PBS (control) or the indicated concentration of NA for 4 hr. (**A–C**) Western blot analyses for whole-cell lysates were performed with an anti-E-cadherin antibody. Membranes were stripped and reblotted for Snail1 and β-actin. Representative images of Western blots are shown in A, and relative intensity of E-cadherin and Snail1 normalized against β-actin was calculated and summarized in (**B,C**), respectively. (**D–F**) Total RNA was extracted, and RT-PCR was carried out for the transcripts of *E-cadherin, snail1*, and *GAPDH*. Representative images of semi-quantitative RT-PCR are shown in (**D**) and relative expression levels of *E-cadherin* and *snail1* (normalized against *GAPDH*), as determined by RT-qPCR, are shown in (**E**,**F**) respectively. NS, not significant; **P < 0.01; ***P < 0.001. (**G–J**) Cells were fixed and processed for DAPI staining (blue) and immunocytochemistry for E-cadherin (green). (**K**) U251 cells were treated with MG132 and NA or PBS (control), IP was carried out for cell lysates with an anti-Snail1 antibody, and Western blot was performed with an anti-ubiquitin antibody. Western blot for whole-cell lysates (WCL) was also performed separately with an anti-Snail1 antibody.

A characteristic of migratory mesenchymal-like cells, as opposed to non-migratory epithelial-like cells, is weaker cell-cell adhesion. We therefore examined if NA treatment of U251 cells affects cell-cell adhesion. Western blot and quantitative RT-PCR (RT-qPCR) analyses reveal that treatment of U251 cells with 3.5 and 7.0 mM NA for 4 hr resulted in upregulated protein and mRNA levels of E-cadherin (Fig. 3A,B,D,E), a major component of the adherens junctions. This was further validated by immunocytochemistry results, which show that there was an increase in E-cadherin signal at the cell-cell boundaries upon NA treatment (Fig. 3G–J). Similarly, tight junction protein ZO-1 also increased drastically (Fig. S3A-S3C), suggesting that overall cell-cell adhesion was enhanced.

EMT is a common mechanism that cells of the epithelial origin utilize to initiate migration during developmental processes and tumor metastasis^21^. A hallmark of EMT is the downregulation of cell-cell adhesion molecules such as E-cadherin and ZO-1. Because glial cells are developmentally derived from the neuroepithelial lineage, and glioma cells are known to undergo EMT-like process to become invasive^22^,^23^, the upregulation of both E-cadherin and ZO-1 indicates that the EMT-like process is inhibited in U251 cells treated with NA. The zinc-finger transcription factor Snail1 is able to recruit several chromatin-modifying enzymes to the *E-cadherin* promoter, thereby epigenetically silencing *E-cadherin* expression in migratory tumor cells^24^,^25^. Although Snail1 does not seem to regulate the transcription of ZO-1, ectopically expressed Snail1 has been shown to reduce ZO-1 protein levels post-transcriptionally^26^. Hence we went on to assess the effects of NA treatment on Snail1 expression. Incubation of U251 cells in increasing concentrations (3.5 and 7.0 mM) of NA for 4 hr resulted in decreasing levels of Snail1 protein (Fig. 3A,C), which correlated well with increasing levels of E-cadherin protein and mRNA (Fig. 3B,E). However, no decrease in *snail1* transcript was detected (Fig. 3D,F), indicating that the regulation of Snail1 by NA is post-transcriptional.

Because Snail1 is known to be degraded through the ubiquitin-proteasome pathway^27^, we asked if NA regulates Snail1 ubiquitination and/or degradation. Pre-treatment of U251 cells with the proteasome inhibitor MG132 abolished the effect of 7.0 mM NA on Snail1 protein levels, and co-immunoprecipitation (co-IP) assays detected elevated levels of ubiquitin associated with Snail1 upon treatment with NA (Fig. 3K). Together these results suggest that NA boosts ubiquitin-proteasome-mediated Snail1 degradation as well as cell-cell adhesion.

**Figure 4:**
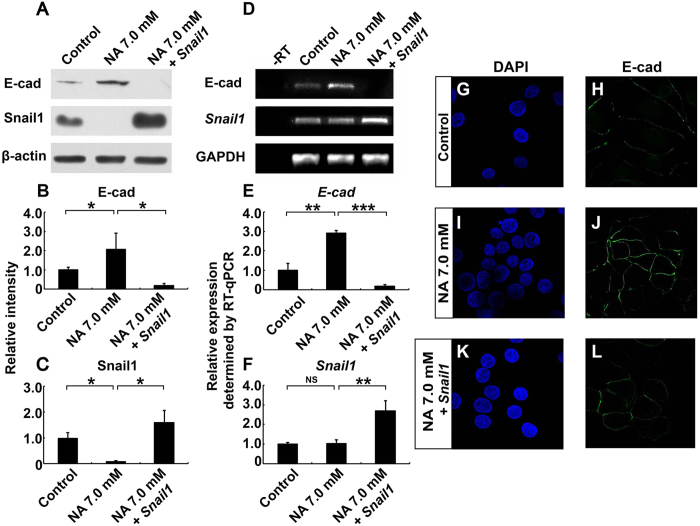
Snail1 reverses the effects of NA on E-cadherin expression. U251 cells were treated with PBS (control) or 7.0 mM NA for 4 hr, or transfected with a plasmid expressing Snail1 and then treated with 7.0 mM NA for 4 hr. (**A**) Western blot analyses for whole-cell lysates were performed with an anti-E-cadherin antibody. Membranes were stripped and reblotted for Snail1 and β-actin. Representative images of Western blots are shown in A, and relative intensity of E-cadherin and Snail1 normalized against β-actin was calculated and summarized in (**B,C**) respectively. (**D–F**) Total RNA was extracted, and RT-PCR was carried out for the transcripts of *E-cadherin, snail1*, and *GAPDH*. Representative images of semi-quantitative RT-PCR are shown in (**D**), and relative expression levels of *E-cadherin* and *snail1* (normalized against *GAPDH*), as determined by RT-qPCR, are shown in E and F, respectively. NS, not significant; *P < 0.05; **P < 0.01; ***P < 0.001. (**G–L**) Cells were fixed and processed for DAPI staining (blue) and immunocytochemistry for E-cadherin (green).

To determine if the regulation of cell-cell adhesion by NA depends on its ability to promote Snail1 degradation, we tested if exogenously overexpressed Snail1 could reverse the increase in E-cadherin level in NA-treated U251 cells. As shown in Fig. 4A–F, overexpression of Snail1 did abrogate the effects of NA on inducing the mRNA and protein expression of E-cadherin. Immunocytochemistry data further confirm the loss of E-cadherin at cell-cell boundaries caused by Snail1 re-expression (Fig. 4G–L). We conclude from the above data that NA upregulates cell-cell adhesion in U251 cells by facilitating the ubiquitination and turnover of Snail1.

### Injection of NA leads to reduced Snail1 levels and delayed neural crest migration in *Xenopus* embryos

**Figure 5:**
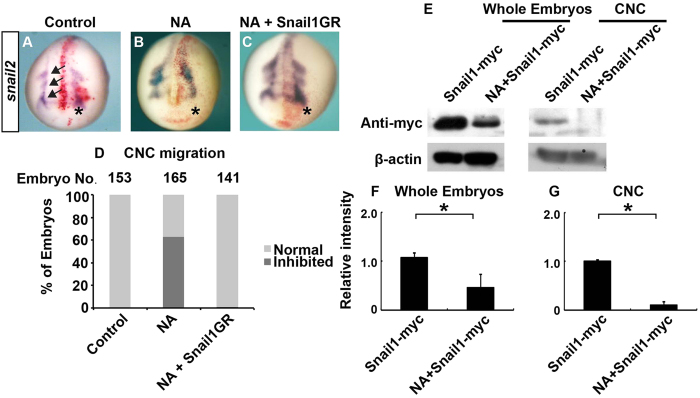
NA inhibits CNC migration in *Xenopus* embryos by inducing Snail1 degradation. (**A–D**) *Xenopus* embryos were injected in one blastomere at 2-cell stage with PBS (control; **A**), NA (70 ng; **B**), or NA combined with mRNA encoding GR-fused Snail1 (**C**). To induce nuclear translocation of Snail1, dexamethasone was added at stage 10–10.5 (**C**). Embryos were cultured to stage ~18 and processed for *in situ* hybridization for *snail2*. The injected side (on the right and denoted with an asterisk) was labeled with co-injected β-galatosidase (red), and arrows indicate the directions of migration of the three CNC streams. A representative embryo from each group is shown in (**A–C**), and quantitative results are summarized in (**D**). (**E**) Embryos were injected with mRNA encoding myc-tagged Snail1, and cultured with or without NA until stage 15–17 (shortly before CNC migration). Western blot was carried out with an anti-myc antibody for whole-embryo lysates or lysates of dissected CNC. Representative images of Western blots are shown in (**E**), and relative intensity of myc-tagged Snail1 normalized against β-actin was calculated and summarized in (**F**) (whole embryos) and (**G**) (CNC). *P < 0.05.

The vertebrate neural crest cells are highly migratory stem cells that are induced at the neural plate border (NPB) during gastrulation. These cells subsequently undergo EMT to delaminate from the closing neural tube, migrate to various destinations and give rise to a wide range of derivatives including neurons and glia of the peripheral nervous system^28^. Similar to cancer cells, migration of neural crest cells from the neural tube also depends on transcription factors that regulate EMT, including Snail1 and Snail2^8^. The *Xenopus* cranial neural crest (CNC) is frequently used as an easily accessible and manipulable *in vivo* model for studying the mechanisms underlying EMT^29^,^30^,^31^,^32^. To test if NA regulates EMT and migration of CNC cells, we injected 70 ng NA into one cell of 2-cell stage *Xenopus* embryos (with a final NA concentration of ~5 mM). *In vitro* transcript of β-galactosidase was co-injected as a lineage tracer, and the uninjected side served as a negative control. The induction of CNC can be subdivided into two steps: the initial formation of the NPB, as indicated by the expression of transcription factors such as Pax3, Zic1 and Msx1 (also called NPB specifiers), and subsequent specification of the CNC, as indicated by the expression of another set of transcription factors such as Snail1 and Snail2 (also called CNC specifiers)^33^,^34^. Notably, some of the CNC specifiers, including both Snail1 and Snail2, also control EMT and CNC migration at later stages (see discussion above). At late gastrula stage (stage ~12) when the CNC had just been induced, we did not observe any effect of NA on the expression of any of the NPB specifiers (*msx1, pax3*, or *zic1*) or CNC specifiers (*snail1* or *snail2*), suggesting that both steps of CNC induction occurred normally (Fig. S4A-S4F). At stage ~18, the CNC had emerged from the neural tube and started to migrate in three separate streams (Fig. 5A). By contrast, in embryos injected with NA, the CNC failed to migrate from the neural tube on the injected side, although it migrated normally on the uninjected side (Fig. 5B,D). At stage ~20 (several hours later), CNC started to migrate in three streams on the injected side (data not shown), probably because NA was metabolized by this time. Thus NA transiently inhibited CNC migration but had no effect on the induction of CNC.

One possible explanation for the delayed CNC migration is that NA decreases the levels of EMT inducers such as Snail1, as we observed in U251 cells *in vitro*. To test this hypothesis, we injected embryos with an mRNA encoding myc-tagged *Xenopus* Snail1, and compared the levels of exogenously expressed Snail1 in embryos with and without co-injected NA. Both whole embryo lysates and dissected CNC showed reduced Snail1 protein levels upon NA treatment (Fig. 5E–G), although the amount of injected Snail1-myc mRNA was the same in embryos with and without NA co-injection. This is consistent with the ability of NA to downregulate Snail1 post-transcriptionally, as we observed in U251 cells (Fig. 3). To further assess if NA interferes with CNC migration via Snail1, we took advantage of an inducible Snail1-glucocorticoid receptor (GR) fusion protein, which resides in the cytoplasm until addition of the GR agonist dexamethasone triggers Snail1 nuclear import^35^. Dexamethasone was added at the beginning of gastrulation (stage 10–10.5) to avoid earlier developmental defects that could be caused by Snail1 overexpression. As shown in Fig. 5C,D, overexpression of Snail1 completely rescued the delayed CNC migration caused by NA. These results suggest that similar to the inhibition of EMT-like process and invasion in U251 cells, NA exerts its effect on CNC migration through regulation of Snail1.

### NA treatment blocks glioma cell invasion and improves survival *in vivo*

**Figure 6:**
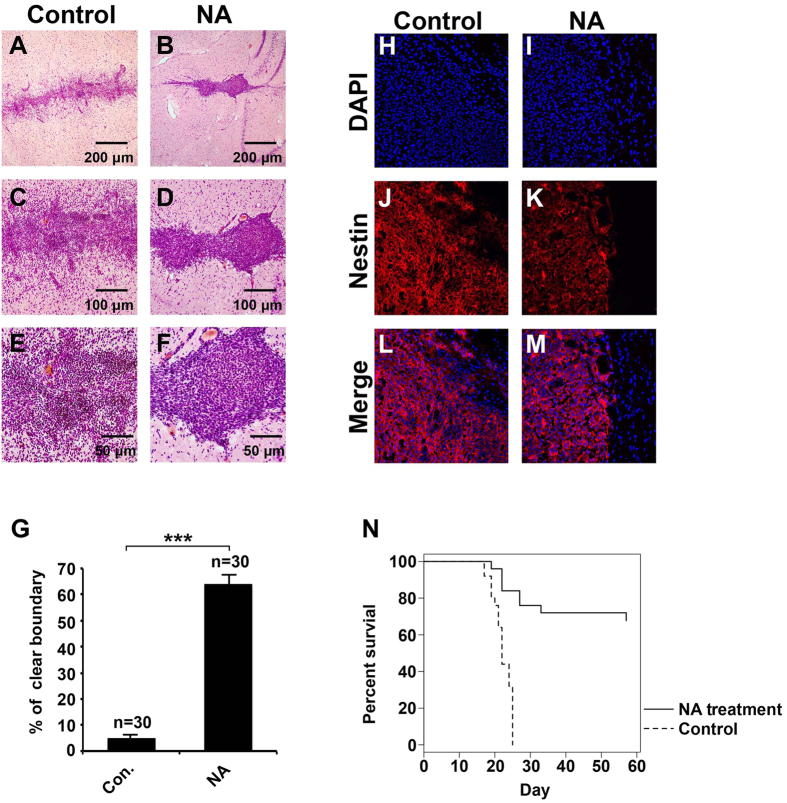
NA injection inhibits C6 glioma cell invasion *in vivo* and improves survival in a rat model of glioma. Rats allografted with C6 cells were injected with PBS (control) or NA as described in Methods. (**A–G**) Brain slices were collected and H&E staining was carried out. Representative images of brain slices collected from rats injected with PBS or NA are shown in (**A–F**) (with indicated magnification), and quantitative results from both groups (6 rats/group) are summarized in G. Arrows indicate C6 glioma cells. ***P < 0.001. (**H–M**) Brain slices were processed for DAPI staining (blue) and immunocytochemistry for Nestin (red). (**N**) NA significantly improved the survival of C6-allografted rats as compared with control (PBS; n = 25 for each group, P < 0.001).

Given the effects of NA on the U251 GBM cells *in vitro* and *Xenopus* CNC *in vivo*, it was tempting to evaluate if NA can block glioma invasion in an animal model. To this end we allografted rats with C6 glioma cells, and conducted on-site NA injections starting from day 4 after grafting. A single dosage of 31 μg NA (5 μl of 50 mM NA) was injected into the allograft everyday for 20 consecutive days. We then sacrificed the rats and assessed the infiltration of tumor cells into normal brain tissues by examining 5 randomly selected allograft regions in brain slices collected from each rat. As compared with normal brain tissues, H&E stained C6 cells displayed nuclear atypia that is characteristic of high-glade glioma (arrows in Figs 6A,B and S5). In the control group injected with PBS, C6 glioma cells frequently infiltrated into normal brain tissues, and only <5% of all chosen regions had a clear boundary between tumor and normal brain (Figs 6A,C,E,G and S5A-S5C). By contrast, in >60% of the chosen regions in C6 allografted rats treated with NA, we observed a clear boundary with fewer tumor cells infiltrating normal brain tissues (Figs 6B,D,F,G and S5D-S5F). The intermediate filament protein Nestin is abundantly expressed in certain high-grade gliomas as well as embryonic cerebrum, but not in normal adult brain tissues^36^,^37^. Our immunohistochemistry results for Nestin show less intermingling between tumor and non-tumor cells in NA-treated brain slices as compared with control (Fig. 6H–M), further confirming that NA inhibits the infiltration of C6 glioma cells into normal brain tissues. Finally, we determined if NA can improve the survival of C6-allografted rats. As shown in Fig. 6N, ~70% of the allografted rats that were continuously administered with NA were still alive on day 58. This is in stark contrast with the control group, which all died by day 24 (Fig. 6N). Thus NA displayed significant beneficial effects on glioma *in vivo*.

## Discussion

Although NA and its derivatives have been intensively studied for decades, new functions and mechanisms of action for them continue to emerge. Recently, we reported that treatment of 3T3 cells with high concentrations of NA leads to dynamic changes in intracellular calcium concentration and disassembly of the cytoskeletal structures^17^. These observations prompted us to test if NA has any effects on malignant glioma cells, which are known for their high invasive activity. We found that treatment with relatively lower concentrations of NA caused loss of mesenchymal phenotype in U251 cells and inhibited glioma invasion *in vitro* and *in vivo*. At the molecular level, NA promoted Snail1 degradation and enhanced cell-cell adhesion, suggesting inhibition of an EMT-like process in glioma cells.

The roles of EMT in glioma cell migration remain controversial, mainly due to the physiological difference between glial and epithelial cells along with the lack of an E-cadherin to N-cadherin switch, which is characteristic of EMT, in glioma cells^38^,^39^. In fact, most malignant glioma cells express very low levels of endogenous E-cadherin (Figs 3 and 4)^39^,^40^,^41^. However, accumulating evidence in the recent years strongly indicates the existence of an EMT-like process, sometimes referred to as “glial-to-mesenchymal transition”, that plays important roles in regulating GBM cell invasion^39^,^40^,^41^. A key observation was that the majority of GBM cells are mesenchymal (Fig. 1)^39^,^40^,^41^, providing an explanation for the lack of E-cadherin expression and high invasive activity of these cells. While multiple EMT regulators are involved in controlling the mesenchymal phenotype of GBM cells, the zinc-finger transcription factor Snail1 is of particular interest. Snail1 expression correlates well with WHO tumor grade, and is elevated in clinically recurrent malignant glioma after treatment with ionizing irradiation^42^,^43^,^44^. Depletion of Snail1 causes reduced expression of mesenchymal markers and loss of mesenchymal phenotype, as well as inhibition of GBM cell invasiveness *in vitro* and *in vivo*^42^,^43^,^45^,^46^,^47^. These effects are accompanied by an increase in E-cadherin level similar to what we observed here with NA treatment^45^,^47^ (Figs 3 and 4), suggesting that E-cadherin expression is normally repressed by Snail1 in malignant glioma, and that such a repression is reversible. Thus at least some malignant glioma cells can be considered as cells that have undergone a reversible EMT-like process to become mesenchymal and invasive, and NA treatment may provide a novel method to reverse this process and inhibit the invasion of these tumor cells.

The molecular mechanisms that regulate neural crest EMT and migration are evolutionarily conserved and highly similar to the mechanisms that regulate cancer EMT and migration/invasion^8^,^48^,^49^. In addition, recent evidence suggests that some types of glioma are neural crest-derived^1^,^2^, making the neural crest an excellent model for studying glioma cell invasion. For instance, a recent study shows that chemical compounds that inhibit CNC migration in *Xenopus* embryos also inhibit GBM cell invasion in mice^50^. In the current study we found that NA promotes degradation of Snail1, a major EMT regulator whose function is evolutionarily conserved^8^,^48^,^49^, in both U251 GBM cells and the *Xenopus* CNC. This finding provides another example of the close relationship between developmental and pathological EMT, and supports the use of non-mammalian vertebrates such as *Xenopus* as simple and more accessible models to identify and characterize drug candidates for cancer therapy. Furthermore, the inhibition of CNC migration by NA in *Xenopus* embryos also implies a potential risk of CNC-related birth defects in babies whose mothers take high dosages of NA during early pregnancy (e.g., for treating cardiovascular diseases).

To better evaluate the therapeutic potential of NA, it is important to understand the mechanism of action for NA in malignant glioma. At this point it is not clear if the inhibition of glioma invasion by NA is related to its ability to elevate intracellular calcium levels, as we reported previously for 3T3 cells^17^, but we did observe transient calcium spikes in U251 cells induced by 3.5 and 7.0 mM NA (data not shown). In addition, activation of either hydroxyl-carboxylic acid receptor 2 (HCA_2_; previously known as GPR109A or HM74a) or TRPV channels, two types of NA receptors, can induce calcium influx^51^,^52^. Current efforts are therefore focused on testing if blocking calcium signaling can interfere with the effects of NA on glioma invasion. The studies of NA as a vitamin in glioma pathology can be traced back to more than half a century ago^53^. Nicotinamide, the amide of NA that has the same vitamin function but does not affect lipid metabolism^54^, has also been shown to have tumor suppressor activity^55^,^56^. However, our preliminary results suggest that nicotinamide does not mimic NA in causing any of the effects on glioma cells that we observed here (data not shown). Moreover, the concentrations of NA that we used to achieve an inhibition of U251 cell invasion *in vitro* (mM level) were much higher than the recommended daily dosage of NA as a vitamin, but comparable to those used for preventing cardiovascular diseases^57^. Thus instead of functioning as a vitamin, NA likely inhibits glioma invasion through specific receptors such as HCA_2_ and/or TRPVs, which do not respond effectively to nicotinamide^19^,^58^. Interestingly, both HCA_2_ and TRPVs have been shown to regulate PI3K/Akt signaling^59^,^60^,^61^, a signaling pathway that is upstream to control GSK-3β-mediated phosphorylation and subsequent ubiquitination of Snail1^62^,^63^. Hence it will be of interest to investigate if NA affects the activation of GSK-3β and/or Akt and, if so, how these effects are related to intracellular calcium levels. Another important question is whether NA could induce the differentiation of glioma stem cells, as reported recently for other anti-cancer drugs such as taxol^64^. Although we did not observe any apparent changes in the level of stemness marker Nestin or differentiation marker β-tubulin with immunostaining (Figs 1 and 6J,K), more quantitative methods such as real-time RT-PCR and Western blot are needed to determine if there are any relatively subtle effects of NA on glioma differentiation.

The inhibition of GBM invasion *in vivo* by NA (Fig. 6) suggests that it can be used prior to surgery to prevent tumor recurrence. Additionally, 5-fluorouracil (5-FU), an antimetabolite that is being actively tested for treatment of malignant glioma^65^, has been reported to cause NA deficiency^66^. This raises an intriguing possibility of using NA in combination with 5-FU for treating malignant glioma. Given that Snail1 is a key EMT regulator in many types of cancer, it is also possible that NA has beneficial effects on other invasive tumors. Indeed, our results suggest that the invasion of malignant melanoma cells is also inhibited by NA (Fig. S2E-S2H), pointing to a general role of NA in regulating tumor invasion.

NA is widely used as an antidyslipidemic drug, and the abundant safety data that are available may greatly facilitate its potential future trials in other diseases such as cancer. We show here that daily injections of 5 μl of 50 mM NA were able to inhibit C6 GBM cell invasion and improve the survival of allografted rats. The overall dosage (31 μg per rat per day) was equivalent to or slightly below the dietary intake reference for NA as a vitamin (14–18 mg per person per day). Therefore, we do not expect any severe side effects on the other parts of the body. However, whether the initial high local concentration of NA could lead to damages to surrounding neurons or glia in the brain remains to be examined. Other potential adverse effects that could be caused by the transient high local concentration of NA include flushing^54^. Flushing is mediated by HCA_2_ and TRPV channels, which may also mediate the anti-tumor activity of NA that we report here. Fortunately, this side effect is not life-threatening and may be acceptable for the treatment of highly deadly diseases such as malignant glioma. In conclusion, our results reveal a novel function of NA in regulating tumor cell invasion, and support the potential application of NA as a therapy for malignant glioma.

## Methods

### Reagents and cells

High-purity (≥99.5%) NA was purchased from Sigma (Cat. # 72309). U251 glioblastoma cells and C6 cells were provided by the Cell Bank of Type Culture Collection of Chinese Academy of Sciences (Shanghai, China). U251 cells were cultured in Dulbecco’s modified Eagle’s medium (DMEM, Hyclone) supplemented with 10% fetal calf serum (Hyclone), and C6 cells in F12K medium (Sigma) supplemented with 15% equine serum and 2.5% fetal bovine serum (FBS; both sera were from Hyclone). Both cells were incubated at 37 °C with 30% humidity and 5% CO_2_.

### Flow cytometry analyses of apoptosis

Cell apoptosis was assessed by using the Annexin-V Apoptosis Detection kit (Becton-Dickinson) and following the manufacturer’s instruction. Fluorescence intensity was measured by a Becton-Dickinson FACSVantageSE flow cytometer. Original data were analyzed by the WinMDI 2.9 software and presented in the form of dot plots, with fluorescein isothiocyanate (FITC)-conjugated Annexin-V as X axis and PI as Y axis.

### Invasion assays

Transwell membranes were precoated with 24 mg/ml matrigel (R&D Systems, USA), and cells were incubated for 8 hr (for U251 and U87) or 5 days (for B16F10). Cells on the top surface of the insert (seeded side) were fixed with methanol, stained with Giemsa solution and counted under a microscope in 5 randomly selected fields (200×). Alternatively, cells on the seeded side were removed with a cotton swab, and cells adhering to the lower surface were fixed, stained and counted.

### Immunocytochemistry

U251 cells were cultured on Lab-Tek chamber slides (Sigma). After treatment with PBS or NA, the cells were fixed with 4% paraformaldehyde and permeabilized with 0.4% Triton X-100 at room temperature. The cells were then blocked with 5% bovine serum albumin (BSA; Sigma) and incubated with the primary antibody at 4 °C overnight. Primary antibodies used were: β-tubulin (BD Transduction Laboratories™ 556321, 1:400), E-cadherin (BD Transduction Laboratories™ 610181, 1:400), and ZO-1 (Abcam ab59720, 1:50). The cells were subsequently incubated with PE or FITC-conjugated secondary antibody (Santa Cruz Biotechnology) at room temperature, and labeled with DAPI (Sigma) to identify cell nuclei^67^. F-actin stress fibers were labeled with Rhodamine phalloidin (Invitrogen Life Technologies R415, 5 units/ml) in PBS for 15 min at room temperature as described^17^. Fluorescence was detected using an Olympus IX81S1F-3 laser confocal scanning microscope.

### RT-PCR

The mRNA levels of target genes were analyzed using semi-quantitative RT-PCR. Total RNA was extracted from U251 cells with an RNA Simple total RNA kit (Tiangen, Beijing, China), and reverse transcription was performed using the M-MLV first strand kit (Invitrogen). Reverse transcription products were then amplified by semi-quantitative PCR using the HotStart Taq master mix kit (Tiangen). Following a “hot start” at 95 °C for 3 min, samples were cycled at 95 °C for 30 s, 55 °C for 30 s, and 72 °C for 20 s. The total numbers of cycles used were: 26 cycles for *E-cadherin*, 28 cycles for *snail1* and 20 cycles for *GAPDH.* The samples were then given a final 5 min extension at 72 °C. RT-qPCR was carried out with the SYBR Green master mix (Thermo Fisher Scientific), and samples were analyzed using a QuantStudio™ 7 Flex Real-Time PCR System. Following a “hot start” at 50 °C for 2 min and 95 °C for 10 min, samples were cycled at 95 °C for 10 min, 60 °C for 30 s, and 72 °C for 30 s for 40 cycles. The samples were then given a final 5 min extension at 72 °C. Primers used for PCR were: 5′-CGAGAGCTACACGTTCACGG-3′ (forward) and 5′-GGGTGTCGAGGGAAAAATAGG-3′ (reverse) for *E-cadherin*, 5′-ACTGCGACAAGGAGTACACC-3′ (forward) and 5′-GAGTGCGTTTGCAGATGGG-3′ (reverse) for *snail1*, and 5′-TGTGGGCATCAATGGATTTGG-3′(forward) and 5′-ACACCATGTATTCCGGGTCAAT-3′ for *GAPDH*.

### Microinjection of *Xenopus* embryos and *in situ* hybridization

*In vitro* fertilization, embryo culturing, preparation of mRNA, microinjection, and whole mount *in situ* hybridization were conducted as described^35^. For each embryo, 70 ng of NA was injected into one blastomere at 2-cells stage^17^. The gluococorticoid receptor (GR) fusion construct for inducible expression of *Xenopus* Snail1 was prepared and injected as described^68^, and nuclear translocation of Snail1 was induced by addition of dexamethasone at stage 10–10.5. For *in situ* hybridization, embryos were collected at desired stages and the probes for *msx1, pax3, zic1, snail1* and *snail2* were used as described previously^68^,^69^,^70^.

### Western blot and IP

Western blot analyses for whole-cell lysates were performed with the following primary antibodies: E-cadherin (1:5000), Snail1 (Santa Cruz sc-28199, 1:2000), and ZO-1 (1:1000). Detection was carried out using HRP-conjugated secondary antibodies and enhanced chemiluminescence substrate (GE Healthcare), and the relative signal intensity was measured using ImageJ. Membranes were stripped and reblotted for β-actin (Sigma-Aldrich A5316, 1:1,000) as a loading control. For IP, U251 cells were treated with 100 nM MG132 (R&D System) for 8 hr, washed 3 times with fresh PBS, and then cultured overnight (8–12 hr) with NA or PBS (control). Cells were subsequently lysed in PBS + 1% NP-40 containing a protease inhibitor cocktail (Roche). Cell lysates were incubated on ice with 0.2 μg anti-Snail1 antibody for 2 hr, followed by a 2-hr incubation with protein A–Sepharose beads. Beads were then washed with ice-cold radioimmunoprecipitation assay buffer. The bound proteins were dissociated by incubating in SDS-PAGE loading buffer at 95 °C for 10 min and subsequently subjected to Western blot analysis. Immunoblotting was performed using the antibody for ubiquitin (Cell Signaling Technology, 1:2,000).

To determine the effect of NA on Snail1 in *Xenopus*, embryos were injected with 70 pg mRNA encoding myc-tagged Snail1 with or without 70 ng NA in each blastomere at 2-cell stage and cultured to stage 15–17. CNC explants were dissected from these embryos as described previously^35^,^71^. Whole-embryos or dissected CNC explants were subsequently lysed in modified TNE lysis buffer (50 mM Tris-HCl (pH 7.4), 150 mM NaCl, 0.5 mM EDTA, and 0.5% Triton X-100) containing protease inhibitor cocktail (Roche), and processed for Western blot with an anti-myc antibody (Sigma-Aldrich C3956, 1:10000)^35^.

### Evaluation of C6 cell invasion in rats

A rat glioma model was established to test the *in vivo* effect of NA. The C6 glioma cells were orthotopically injected to the right striatum of Sprague-Dawley (SD) rat brains through a pre-settled stainless steel tube. NA and control (PBS) were also injected through the tube. When the treatment was completed, the rats were sacrificed by anesthesia overdose and their brain tissues removed and embedded immediately in optimal cutting temperature compound. The brain tissues were subsequently cut into slices (7-μm thick) with a Leica CM1850 cryostat microtome. Brain slices were fixed with 95% ethanol and stained with hematoxylin and eosin (both were from Sigma). For immunohistochemistry, brain slices were fixed with 4% paraformaldehyde for 30 min, followed by blocking with 5% BSA for 1 hr at room temperature. The slices were then incubated with rabbit anti-Nestin polyclonal antibody (Millipore) at 4 °C overnight, washed, incubated again with phycoerythrin-conjugated secondary antibody (Santa Cruz Biotechnology) for 45 min at room temperature, and stained with DAPI to label nuclei. After being sealed with neutral mounting medium (Jiangyuan, Jiangsu, China), the slices were observed under an Olympus upright microscope.

### Ethics statement

Methods involving live animals were carried out in accordance with the guidelines and regulations enacted and enforced by Chinese National Ministry of Science and Technology as well as National Ministry of Health. All experimental protocols were approved by the Institutional Lab Animal Ethics Committee at Kunming University of Science and Technology, Kunming, China.

## Additional Information

**How to cite this article:** Li, J. *et al*. Nicotinic acid inhibits glioma invasion by facilitating Snail1 degradation. *Sci. Rep.* **7**, 43173; doi: 10.1038/srep43173 (2017).

**Publisher's note:** Springer Nature remains neutral with regard to jurisdictional claims in published maps and institutional affiliations.

## Supplementary Material

Supplementary Dataset 1
