# Supplementary material for: Nicotinic acid inhibits glioma invasion by facilitating Snail1 degradation
Source: Sci Rep. 2017 Mar 3;7:43173. doi: 10.1038/srep43173 (PMC5335718; doi:10.1038/srep43173)
Supplement: Supplementary Dataset 1 [file srep43173-s1.doc]

**Nicotinic acid inhibits glioma invasion by facilitating Snail1 degradation**

Jiejing Li^1, †, *^, Jiagui Qu^1, †^, Yu Shi^2, †^, Mark Perfetto^3, 4, †^, Zhuxian Ping^1^, Laura Christian^3^, Hua Niu^1^, Shuting Mei^5^, Qin Zhang^1^, Xiangcai Yang^1^, Shuo Wei^3, 4, *^

^1^ Department of Clinical Laboratory, The Affiliated Hospital of KMUST, Medical School, Kunming University of Science and Technology, Kunming 650032, China.

^2^ Department of Clinical Laboratory, Children’s Hospital of Chongqing Medical University, Chongqing, China; Ministry of Education Key Laboratory of Child Development and Disorders; Chongqing Key Laboratory of Pediatrics; Chongqing Key Laboratory of Translational Medical Research in Cognitive Development and Learning and Memory Disorders, Chongqing 400014, China.

^3^ Department of Biology, West Virginia University, Morgantown, WV 26506, United States.

^4^ Department of Biological Sciences, University of Delaware, Newark, DE 19716, United States.

^5^ Department of Gerontology, First People’s Hospital of Yunnan Province, Kunming 650032, China.

^†^ Equal contributors.

* Correspondence to Jiejing Li, [jjli@kmust.edu.cn](mailto:jjli@kmust.edu.cn) or Shuo Wei, [swei@udel.edu](mailto:swei@udel.edu)


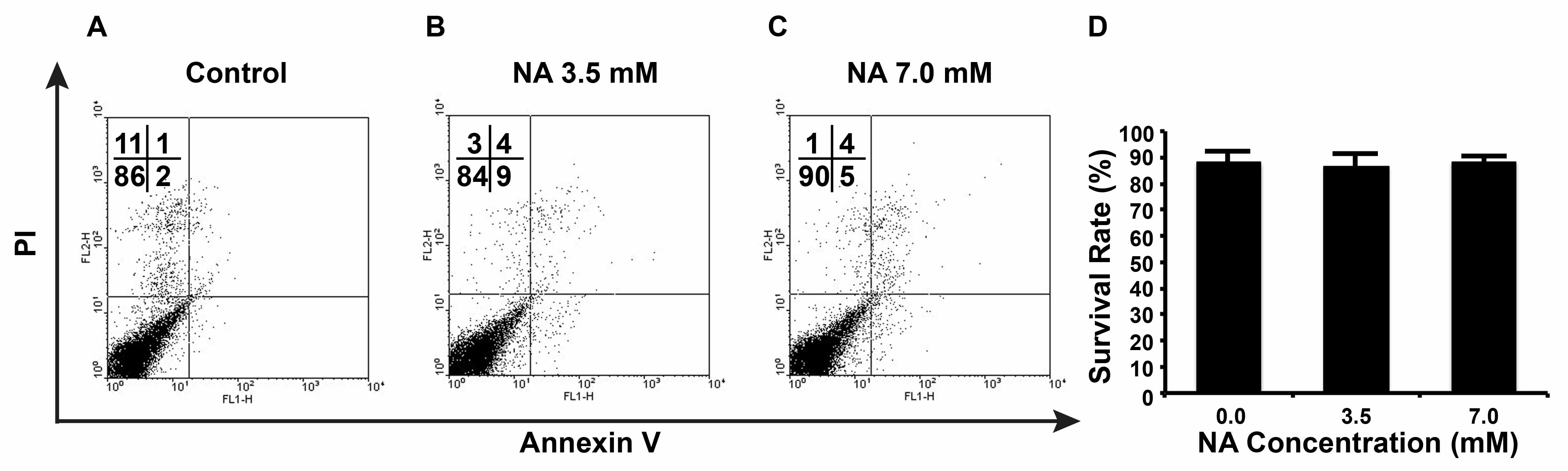


**Supplemental Fig. S1. Apoptosis assays for U251 cells treated with NA.** U251 cells were cultured in the indicated concentration of NA for 20 hr. Cells were harvested and stained with Annexin V and PI, and cell sorting was carried out as described in Methods. Profiles of cells analyzed in a representative experiment are shown in A-C, with low Annexin V and low PI staining (lower left square) indicating live cells. Results of 3 independent experiments are summarized in D.


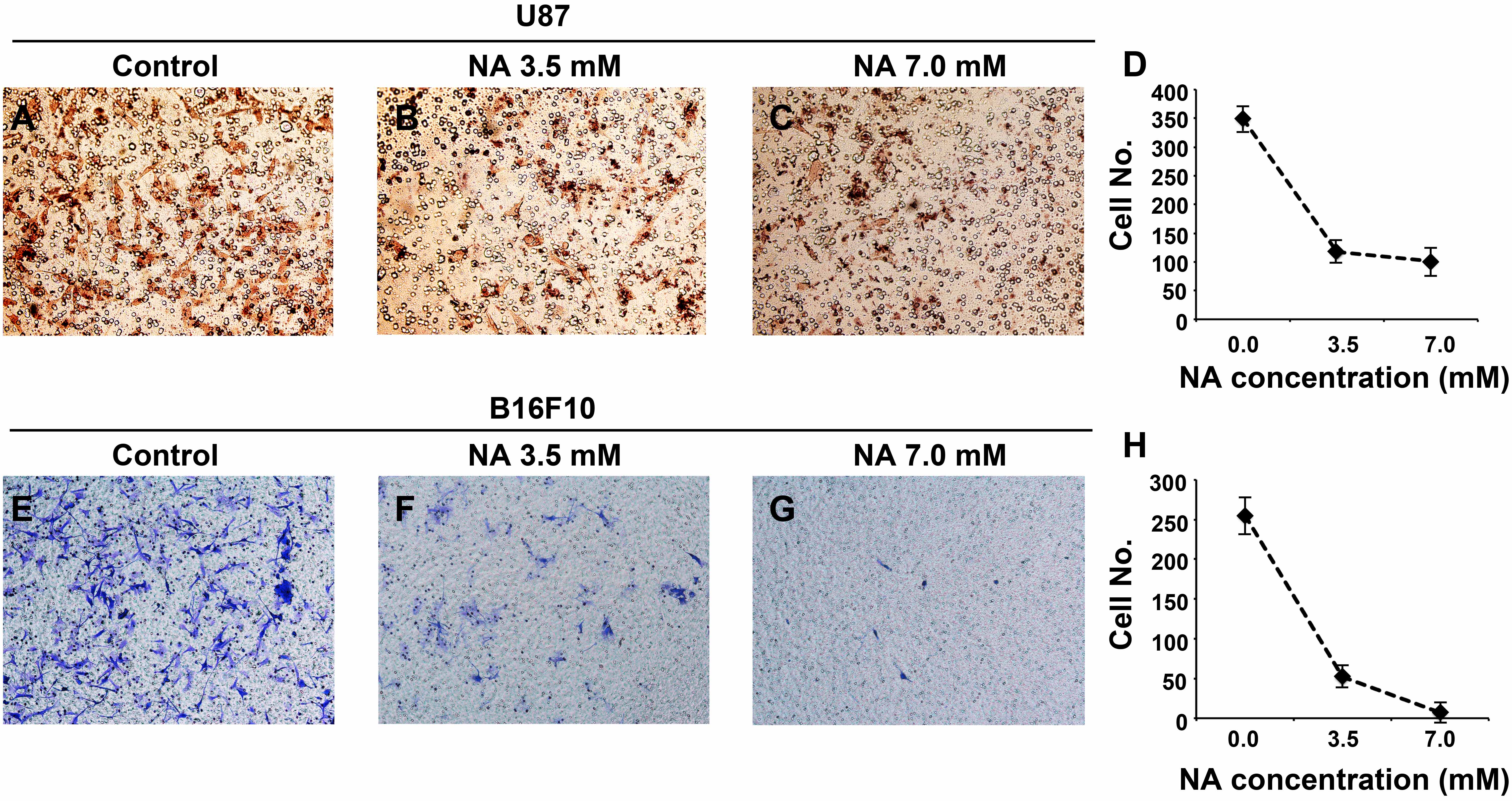


**Supplemental Fig. S2. NA inhibits the invasion of U87 and B16F10 cells.** Transwell assays were carried out for U87 GBM cells or B16F10 melanoma cells incubated in PBS (control) or the indicated concentration of NA, as described in Methods. Images of cells (stained with Giemsa) that invaded through the matrigel in a representative experiment are shown, and results of 15 different regions in 3 independent experiments (5 regions per experiment) are summarized in D and H for U87 and B16F10 cells, respectively.


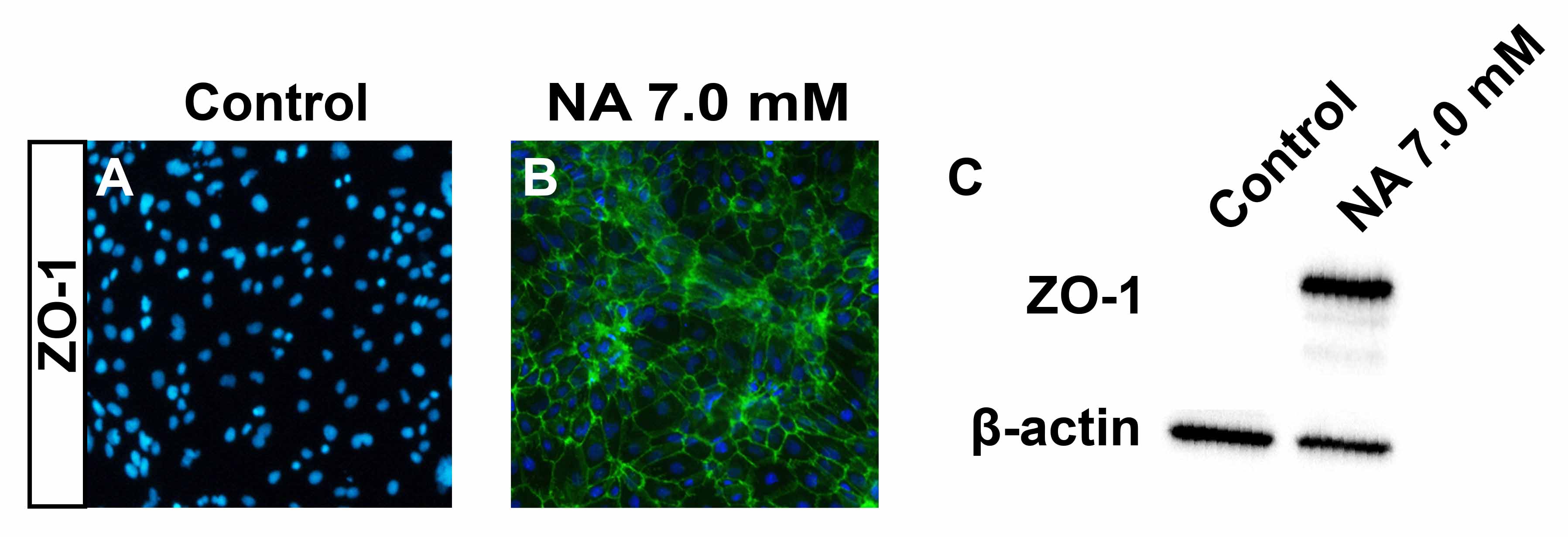


**Supplemental Fig. S3. Effects of short-term NA treatment on ZO-1.** U251 cells were treated with PBS (control) or 7.0 mM NA for 4 hr. (A and B) DAPI labeling for nuclei (blue) and immunocytochemistry for ZO-1 (green) were carried out as described in Methods, and images taken from blue and green channels were merged. (C) Western blot for whole-cell lysates with an anti-ZO-1 antibody.

**
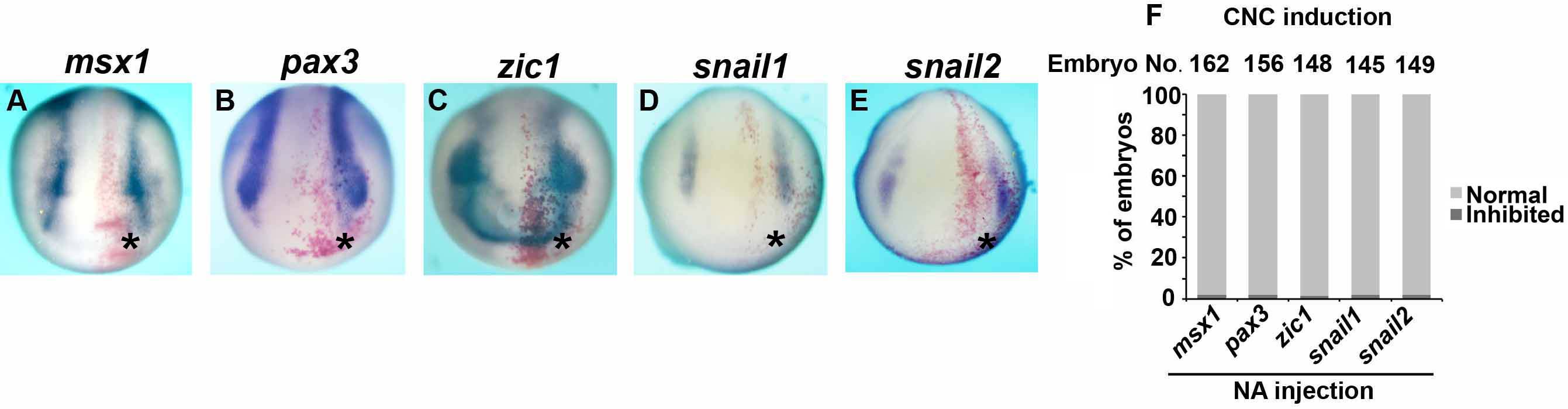
**

**Supplemental Fig. S4. NA does not affect *Xenopus* CNC induction.** *Xenopus* embryos were injected in one blastomere at 2-cell stage with 70 ng NA, cultured to stage ~12, and processed for in situ hybridization for *msx1* (A), *pax3* (B), *zic1* (C), *snail1* (D) or *snail2* (E). The injected side (on the right and denoted with an asterisk) was labeled with co-injected β-galatosidase (red). A representative embryo from each group is shown in A-E, and quantitative results are summarized in F.


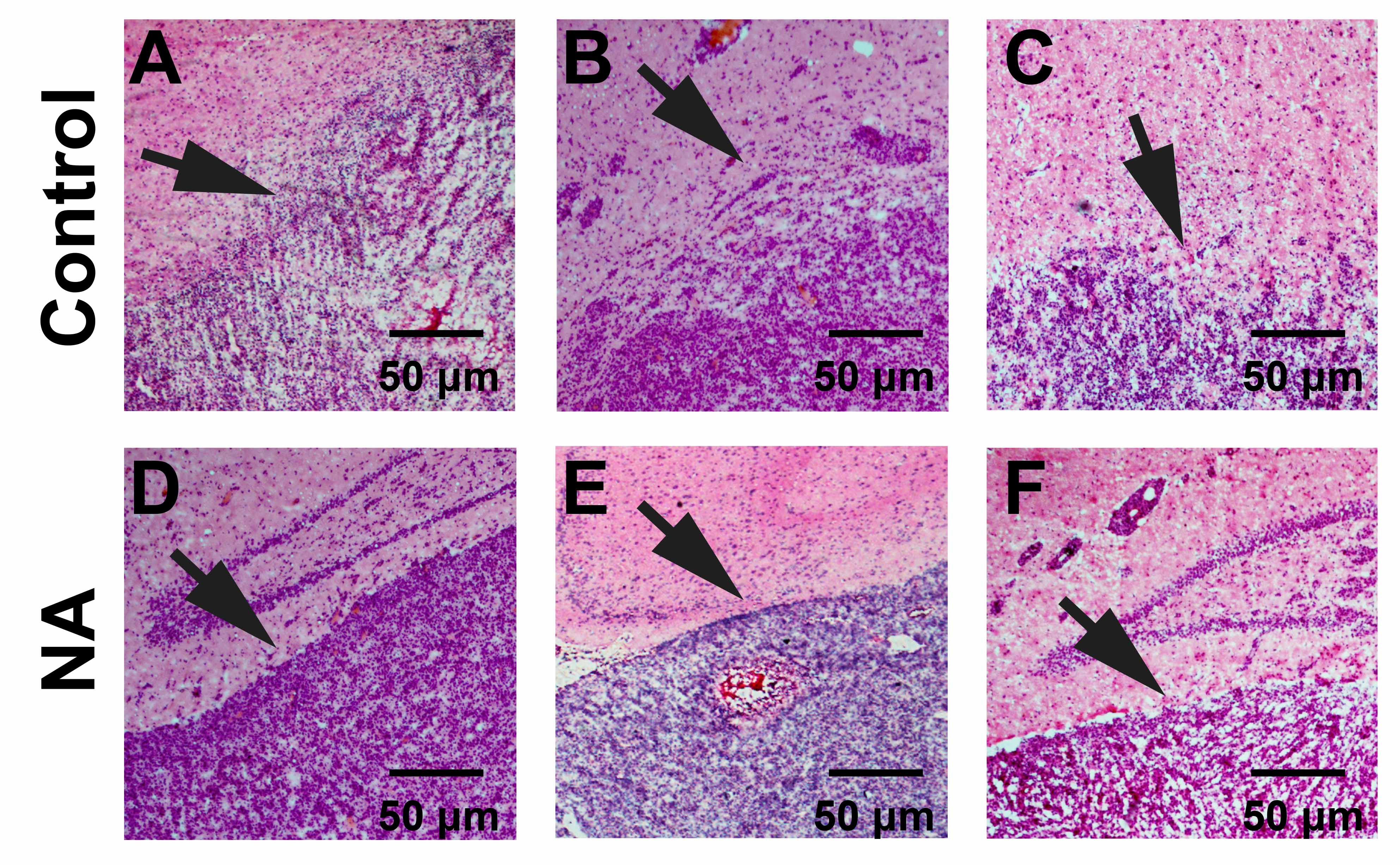


**Supplemental Fig. S5. NA injection inhibits C6 glioma cell invasion *in vivo*.** Rats allografted with C6 cells were injected with PBS (control; A-C) or NA (D-F) as described in Methods. Brain slices were collected and processed for H&E staining, and three representative images of randomly selected slices from each group are shown here. Arrows indicate C6 glioma cells.
